# Diversity of Plant Methionine Sulfoxide Reductases B and Evolution of a Form Specific for Free Methionine Sulfoxide

**DOI:** 10.1371/journal.pone.0065637

**Published:** 2013-06-12

**Authors:** Dung Tien Le, Lionel Tarrago, Yasuko Watanabe, Alaattin Kaya, Byung Cheon Lee, Uyen Tran, Rie Nishiyama, Dmitri E. Fomenko, Vadim N. Gladyshev, Lam-Son Phan Tran

**Affiliations:** 1 Signaling Pathway Research Unit, RIKEN Center for Sustainable Resource Science, Yokohama, Japan; 2 Division of Genetics, Brigham and Women’s Hospital and Harvard Medical School, Boston, Massachusetts, United States of America; 3 National Key Laboratory of Plant Cell & Biotechnology and Agriculture Genetics Institute, Vietnamese Academy of Agricultural Science, Hanoi, Vietnam; 4 Department of Biochemistry and Redox Biology Center, University of Nebraska-Lincoln, Lincoln, Nebraska, United States of America; Instituto de Biociencias - Universidade de São Paulo, Brazil

## Abstract

Methionine can be reversibly oxidized to methionine sulfoxide (MetO) under physiological conditions. Organisms evolved two distinct methionine sulfoxide reductase families (MSRA & MSRB) to repair oxidized methionine residues. We found that 5 MSRB genes exist in the soybean genome, including GmMSRB1 and two segmentally duplicated gene pairs (GmMSRB2 and GmMSRB5, GmMSRB3 and GmMSRB4). GmMSRB2 and GmMSRB4 proteins showed MSRB activity toward protein-based MetO with either DTT or thioredoxin (TRX) as reductants, whereas GmMSRB1 was active only with DTT. GmMSRB2 had a typical MSRB mechanism with Cys121 and Cys 68 as catalytic and resolving residues, respectively. Surprisingly, this enzyme also possessed the MSRB activity toward free Met-*R*-O with kinetic parameters similar to those reported for f*R*MSR from *Escherichia coli*, an enzyme specific for free Met-*R*-O. Overexpression of GmMSRB2 or GmMSRB4 in the yeast cytosol supported the growth of the triple MSRA/MSRB/f*R*MSR (Δ3MSRs) mutant on MetO and protected cells against H_2_O_2_-induced stress. Taken together, our data reveal an unexpected diversity of MSRBs in plants and indicate that, in contrast to mammals that cannot reduce free Met-*R*-O and microorganisms that use f*R*MSR for this purpose, plants evolved MSRBs for the reduction of both free and protein-based MetO.

## Introduction

Among the 20 common amino acids, methionine (Met) is among the most susceptible to oxidation by reactive oxygen species (ROS). Under elevated ROS levels, free and protein-based Met are converted to methionine sulfoxide (MetO), which occurs in a diastereomeric mixture of methionine-*S*-sulfoxide (Met-*S*-O) and methionine-*R*-sulfoxide (Met-*R*-O) [1]. Several proteins have been reported, in which oxidation of Met residues is linked to protein dysfunction or aggregation (reviewed in [2], [3]). Oxidation of Met was also described in signaling proteins, modulating their functions [1], [4]. To repair oxidized Met in proteins, organisms evolved two enzyme families, methionine sulfoxide reductase A (MSRA) that reduces Met-*S*-O and methionine sulfoxide reductase B (MSRB) that reduces Met-*R*-O. It was reported that MSRAs can act on both protein-based and free Met-*S*-O, whereas MSRBs are inefficient against free Met-*R*-O because of extremely low affinity for this substrate [5]–[8]. Lee *et al*. showed that mammalian cells are unable to use Met-*R*-O as a source of Met to support growth [9] and it was also found that yeast cells carrying only *MSRB* gene (with other *MSR*s deleted) failed to grow in both liquid and solid media containing Met-*R*-O as the sole source of Met [10]. Recently, a new enzyme family unique to some unicellular organisms capable of reducing free Met-*R*-O (f*R*MSRs) was characterized [10], [11]. As reported by Lin *et al.*, the f*R*MSR from *E. coli,* which contains a GAF domain, reduced Met-*R*-O with the *K*_m_ of 3,900 µM [11]. Following this study, a yeast homolog of *E. coli* f*R*MSR was characterized with the *K_m_* of 230 µM. It should be noted that due to the nature of the discontinuous assay (where NADPH may become limited at high substrate concentrations), this *K*_m_ might not represent a true value [10].

The catalytic mechanisms of 2-Cys MSRBs and f*R*MSRs are similar to that of MSRAs and involve transient formation of a sulfenic or selenic acid intermediates on the catalytic Cys or selenocysteine [12], [13], which subsequently condenses with a resolving Cys to form a disulfide or selenosulfide bond [10], [11], [14]. 1-Cys MSRBs, such as human MSRB2 and MSRB3 and *A. thaliana* plastidic MSRB1, which do not possess resolving Cys residues, can be reduced *in vitro* by thioredoxin (TRX) by the direct reduction of the sulfenic acid intermediate [15]–[17]. Glutaredoxin was shown to serve as a possible alternative reducing system [18]–[20].

*In vivo* modulation of MSR activities has been reported in yeast [10], [21], fruit fly [22] and mammals [23], which in turn affected resistance to oxidative stress and lifespan. In plants, MSR activities were identified many years ago [24], but their functional characterization has not been carried out until recently [8], [25], [26]. In an attempt to understand the importance of Met oxidation and MetO reduction in soybean’s defense against biotic and abiotic stresses, we carried out a comprehensive characterization of its MSRBs. An exhaustive search of the genome identified 5 members of the *GmMSRB* family. We analyzed their expression profiles in various tissues under normal and drought stress conditions, and characterized their enzymatic properties as well as their roles in protecting against oxidative stress using yeast. Interestingly, characterization of their enzymatic properties revealed that GmMSRB2 could reduce free Met-*R*-O as efficiently as yeast f*R*MSR. Expression of some of the *GmMSRB* genes in the Δ*3MSR* mutant yeast restored the ability to use free-Met-*R*-O as a source of Met to support growth, indicating that soybean MSRBs function in the reduction of both free and protein-based Met-*R*-O.

## Materials and Methods

### In Silico Analysis of GmMSRBs

Using *Arabidopsis MSRB*s as seed sequences, *GmMSRBs* were identified by reciprocal BLAST, and genes were further examined by manual inspection. Full-length sequences containing natural stop codons were used for further analyses. Multiple sequence analyses were done with MEGA4 [27]. Synteny analysis was performed using the online locus search (http://chibba.agtec.uga.edu/duplication/index/locus).

### Soybean Growth, Stress Treatment and Sample Collection

Stress treatment and sample collection of young soybean seedlings were performed as previously described [28]. Drought treatment of V6 vegetative soybean plants (28 days after sowing, containing 6 fully developed trifoliate leaves) was carried out by withholding plants from watering, and sample collection was performed exactly as described previously [29], [30]. Collected samples were quickly frozen in liquid nitrogen and stored at −80°C until use.

### RNA Extraction, cDNA Synthesis and Transcript Analyses by Quantitative PCR (qPCR)

Tissue samples were ground into fine powder using pestle and mortar, and TRIZOL reagent (Invitrogen) was used to isolate total RNA. Total RNA was then treated with Turbo DNA-free DNAse I (Ambion) and subsequently used for first stranded cDNA synthesis. All steps were performed as described [28], [29]. For transcription profiling of *GmMSRB* genes in soybean, primers were designed using Primer3 [31]. Primer specificity was confirmed by BLAST against the soybean genome. For normalization, primers specific for genes encoding F-box and 60S were used as described previously [32]. qPCR was performed as previously described, including data calculation [28].

### Statistical Analysis of Data

qPCR was performed on 3 biological replicates for each treatment, and mean values and standard errors were used for data presentation. For comparison of two mean values, a Student’s *t*-test was applied. All differences with *p*-values less than 0.05 were considered statistically significant. To compare expression of genes with alternative splicing, the sums of primary and secondary transcripts were used.

### Gene Cloning and Site-directed Mutagenesis

Coding sequences of *GmMSRBs* were cloned from the soybean cDNA pool extracted from various tissues and under various treatments using primers listed in Table S1. For construction of expression vectors in yeast, blunt-ended PCR products were first ligated into the pKS vector and sequenced. Correct inserts were excised using *Spe*I and *Sal*I restriction enzymes (see Table S1) and ligated into the p425-GPD vector. To create yeast expression vectors carrying *GmMSRB1* and *GmMSRB4* that do not encode signal peptides, the coding sequences were PCR-amplified from pDEST17 plasmids using primers shown in Table S1, digested with *BamH*I/*Xho*I (for *GmMSRB1*) or *Nde*I/*Xho*I (for *GmMSRB4*) and ligated into appropriately cut p425-GPD vector.

For production of recombinant proteins in *E. coli*, sequences coding for full-length proteins were PCR-amplified from pKS plasmids and ligated into pENTR D/TOPO. The pENTR plasmids carrying correct sequences were recombined into pDEST17 vectors using Gateway® technology. Initial expression analysis showed that full-length GmMSRB1 and GmMSRB4 proteins were not soluble; therefore, constructs that overexpressed proteins lacking the predicted signal peptides were prepared. For this purpose, coding sequences of N-terminal truncated GmMSRB1 & GmMSRB4 (without signal peptides) were PCR-amplified from respective pDEST17 plasmids and inserted into pET21b. For purification of yeast TRX2 (YGR209C) and GRX4 (YER174C) recombinant proteins, *TRX2* and *GRX4* were PCR-amplified using primers listed in Table S1 and inserted into pET15b and pET21b, respectively. Site-directed mutagenesis was performed following the Quickchange® protocol using primers listed in Table S1.

### Protein Expression and Purification

pET21b, pDEST17 and pET15b carrying appropriate coding sequences were transformed into *E. coli* BL21 (DE3) T7 Express® (New England Biolabs) and cultured in media containing recommended concentrations of antibiotics. Protein expression was induced by the addition of isopropyl β-D-1-thiogalactopyranoside (IPTG) to achieve a concentration of 100 µM. Induction of protein synthesis was conducted at 30°C for 4 hours, and the cells were harvested by centrifugation [33]. Purification of His-tag recombinant proteins was performed essentially as described [7], [34].

### Measurements of Methionine Sulfoxide Reductase Activities

TRX-dependent MSR activities were measured by monitoring NADPH consumption as described by Tarrago *et al.* [7]. Briefly, the reaction was initiated by the addition of 200 µM NADPH to the reaction mixture containing 2 µM TRX reductase, 25 µM yeast TRX2, 1–5 µM GmMSRB proteins and variable amounts of MetO or *N*-acetyl-MetO. For the GRX-reducing system, the reaction mixture contained 400 µM NADPH, 0.5 unit yeast glutathione reductase (Sigma), 10 mM GSH, 5 µM yeast GRX4 and 1 mM MetO or *N*-acetyl-MetO. DTT-dependent MSR activities toward dabsyl-MetO or free MetO were also determined using published procedures [10], [35].

### Yeast Complementation and Oxidative Stress Tolerance Assays

A triple yeast mutant strain, whose all 3 *MSRs* (*MSRA/MSRB/fRMSR*) were knocked out, was transformed with p425-GPD plasmids expressing soybean *MSRBs* or yeast *MSRBs/fRMSR* genes under the control of a strong promoter [36]. For complementation assays, the recombinant strains were grown in synthetic media without *L*-Leu, *L*-Met and with the addition of *L*-MetO (20 mg.L^−1^). For oxidative stress protection assay, strains were grown in selective liquid media until the OD_600_ reached 0.6. Subsequently, H_2_O_2_ was added to achieve a final concentration of 2 mM and the treatment was continued for 60 minutes. Cells were washed, diluted and spotted on agar plates.

## Results and Discussion

### Identification and in Silico Analysis of Soybean GmMSRB Genes

**Figure 1:**
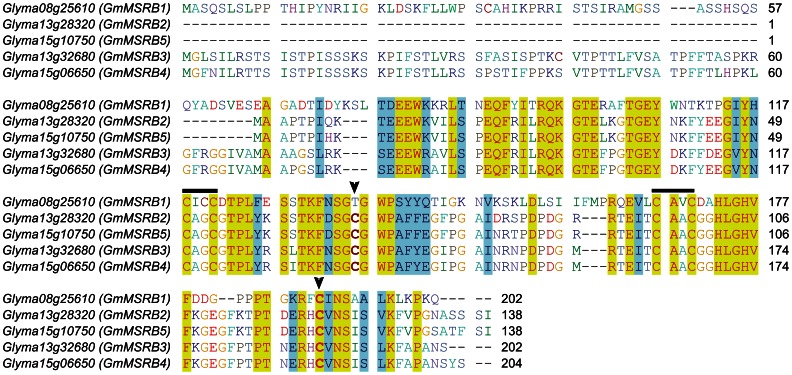
Multiple sequence alignment of GmMSRB proteins. Arrows indicate predicted resolving and catalytic Cys residues, respectively. Black bars indicate two CxxC motifs coordinating a zinc atom. Residues identical in all the 5 sequences were colored red and shaded in yellow, whereas similar residues were shaded in blue.

Although soybean is a palaeopolyploid, its genome possesses only 5 genes encoding MSRBs (*GmMSRBs*), the same number as in rice, poplar and grapevine, but fewer than in *Arabidopsis* (9 *MSRB* genes) [8], [37]. The GmMSRB proteins contain a SelR domain with catalytic and resolving Cys residues predicted to be at positions 121 and 68, respectively (numbering follows the GmMSRB2 sequence) (Table 1 and Fig. 1). Apart from the conserved catalytic Cys, GmMSRBs possess two other conserved CxxC motifs, which apparently coordinate a zinc atom as previously shown for fruit fly and other MSRBs [35]. We found that GmMSRB1 did not possess a resolving Cys residue (Fig. 1). Further analysis with *TargetP* [38] revealed that 3 GmMSRBs (GmMSRB1, GmMSRB3 and GmMSRB4) had signal peptides targeting these proteins to chloroplast. In addition, synteny analysis suggested that 4 *GmMSRB* genes were the result of segmental duplication, including one pair formed by *GmMSRB2* and *GmMSRB5* and another pair formed by *GmMSRB3* and *GmMSRB4* (Fig. S1). Among the 5 *GmMSRB* genes identified, alternative splicing was identified for *GmMSRB2* and *GmMSRB4*. The secondary transcript of *GmMSRB2* (named as *GmMSRB2.2*) encodes a protein lacking the first 29 residues present at the N-terminus of *GmMSRB2.1*. The secondary transcript of *GmMSRB4* (*GmMSRB4.2*) encodes a protein lacking the last 45 residues, including the catalytic Cys residue (Fig. S2).

**Table 1:** Domain feature and signal peptide prediction of soybean MSRB proteins.

Names	Gene ID	E values^a^	Position of catalytic Cys	cTP^b^	mTP	SP	Other	Location^c^	Reliability^d^	TPlength
GmMSRB1	Glyma08g25610	1.0E-48	[190]	0.869	0.061	0.02	0.195	C	2	45
GmMSRB2	Glyma13g28320	6.0E-52	[121]	0.133	0.083	0.11	0.854	–	2	–
GmMSRB3	Glyma13g32680	1.1E-51	[189]	0.971	0.088	0.01	0.038	C	1	67
GmMSRB4	Glyma15g06650	1.1E-51	[189]	0.956	0.102	0.01	0.056	C	1	67
GmMSRB5	Glyma15g10750	1.2E-52	[121]	0.132	0.071	0.1	0.871	–	2	–

a E values for SelR domain prediction by PFAM.

b Target prediction by TargetP.

c Locations: C, Chloroplast; -, not known.

d Reliability score scale from 1 to 5, lower values have higher probability.

### Expression of GmMSRBs in Various Tissues Under Normal and Stress Conditions

**Figure 2:**
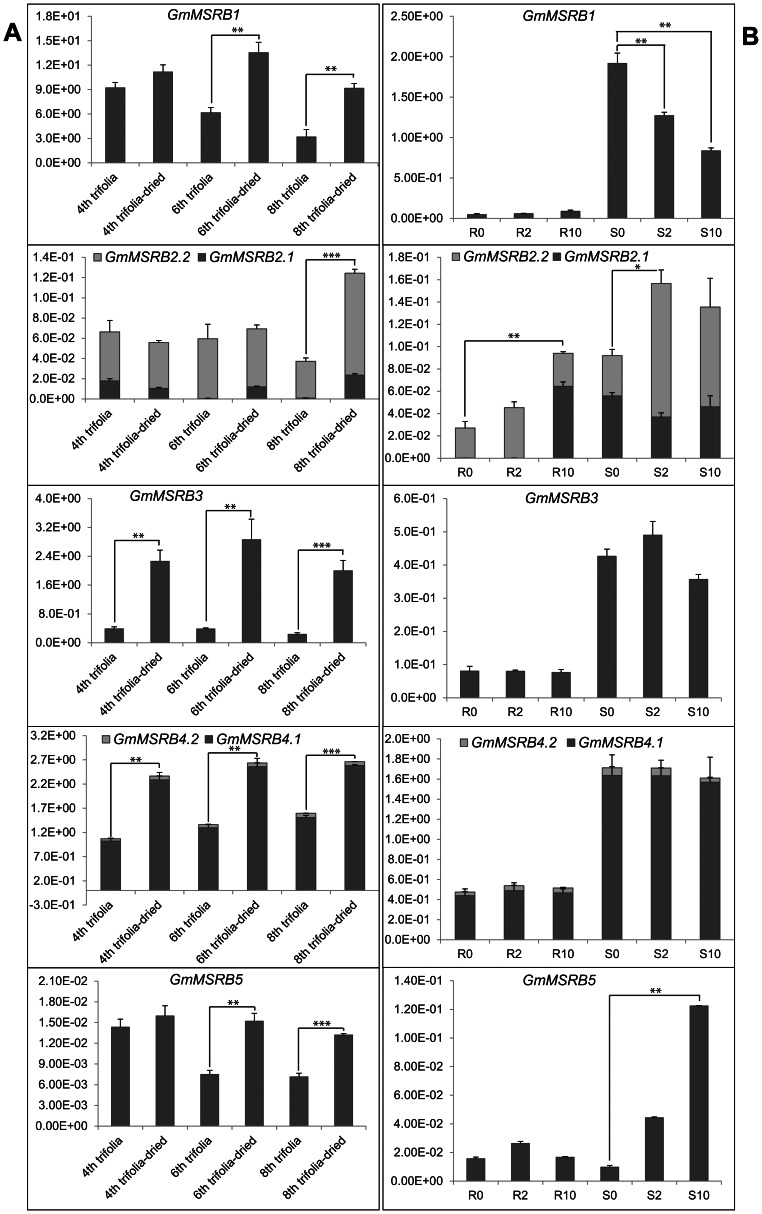
Steady-state transcript abundance (in arbitrary units) of *GmMSRB* genes under normal and drought conditions. (A) Transcript levels in V6-vegetative-stage leaves, (B) transcript levels in roots and shoots of young seedlings; R0, R2, R10 and S0, S2, S10 represented roots (R) or shoots (S) at 0, 2 or 10 h, respectively, under dehydration stress treatment.

To obtain insights into biological functions of *GmMSRBs* under normal and abiotic stress conditions, we analyzed their expression profiles. *GmMSRB1* and *GmMSRB3* were highly expressed in various tissues, especially in the aerial parts, reaching highest levels in leaf (Fig. S3) [39]. Although *GmMSRB3* and *GmMSRB4* formed a duplicated pair, their expression levels were significantly different as judged by steady-state transcript abundance. To gain insight into how these genes function under abiotic stresses, we quantified the steady-state levels of their transcripts (including their spliced forms) using qPCR in V6 vegetative-stage trifoliate leaves, young seedling roots and shoots under normal and dehydration conditions. Results shown in Fig. 2 confirmed the occurrence of alternative splicing in *GmMSRB2* but the data were ambiguous for *GmMSRB4.* Because *GmMSRB4.2* is predicted to be of very low abundance and encodes a predicted protein lacking its catalytic residue, we consider that the presence of this transcript was due to splicing error. The data shown in Fig. 2 also indicated that under drought conditions, the expression of all *GmMSRB*s was induced in the V6-stage leaves, and this effect was more pronounced in younger trifoliate leaves (Fig. 2A). In young seedling roots and shoots, *GmMSRB*s were less responsive to dehydration stress with the exception of *GmMSRB2*, whose expression was upregulated in both roots and shoots, and *GmMSRB5* whose expression was induced only in the shoots (Fig. 2B).

### *In vitro* and *in vivo* Activities of GmMSRB Proteins

**Figure 3:**
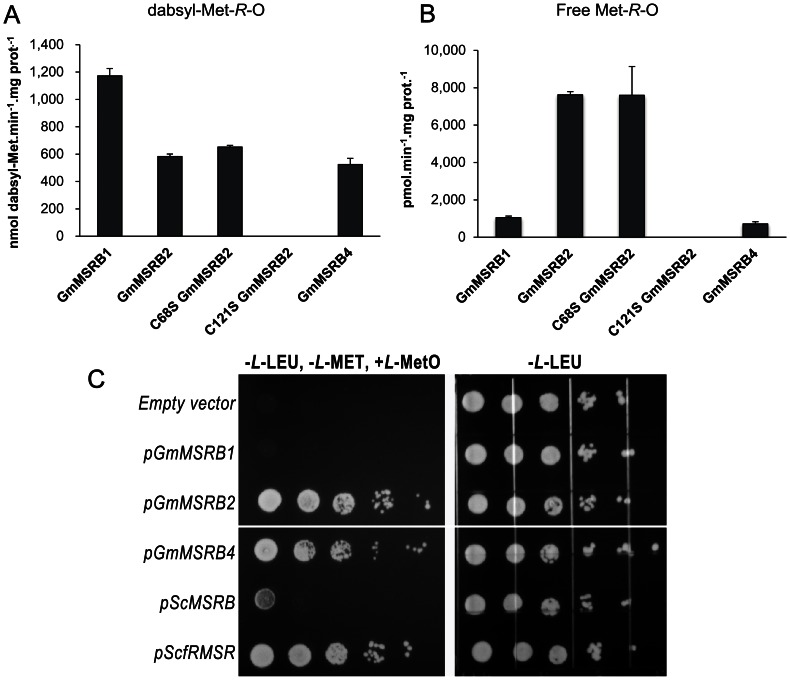
*In vitro* and *in vivo* activities of GmMSRBs. MSR activities of GmMSRBs using DTT as electron donor toward free Met-*R*-O (A) or dabsyl-Met-*R*-O (B). Data presented are the means ±SE of 3 replicates. (C) *In vivo* complementation assay of GmMSRBs. Yeast strain lacking *MSRA, MSRB* and *fRMSR* was transformed with indicated plasmids and grown under selective media (right panel) or selective media plus MetO as the sole source of Met (left panel). Experiment was performed in triplicate and representative data are shown. pGmMSRB1 and pGmMSRB4 did not include sequences encoding signal peptides.

To characterize the function of GmMSRBs, we selected GmMSRB1, GmMSRB2 and GmMSRB4 as representatives. Since GmMSRB3 and GmMSRB5 were duplicated members of GmMSRB4 and GmMSRB2 with very high homology (90 and 95% identity in amino acid sequence, respectively, Fig. 1 and Fig. S1), they likely function very similarly to the corresponding paralogs. The purified GmMSRB proteins were assayed for MSR activities with either DTT or yeast TRX system as reductants. As shown in Fig. 3, in the reaction mixtures containing equivalent amounts of enzymes, GmMSRB1 was two-fold more efficient than GmMSRB2 or GmMSRB4 in reducing dabsylated Met-*R*-O (Fig. 3A). Although MSRBs are known to have activity only for protein-based MetO, several reports suggested that the enzymes may have very low activity with free MetO [5], [7], [8]. This possibility prompted us to assay GmMSRB proteins for their activities toward free MetO. Surprisingly, we found that GmMSRB2 exhibited a high MSR activity with free Met-*R*-O, as this protein released 7621 pmole of Met per minute per milligram protein (Fig. 3B). This activity was 7- and 10- fold higher than those of GmMSRB1 and GmMSRB4, respectively. As oxidized MSRs can be regenerated by TRX or GRX [18], [19], [40], we also assayed the soybean enzymes using yeast TRX and GRX as reduction systems. The data presented in Table 2 show that GmMSRB1 did not exhibit MSR activity with yeast TRX2 or GRX4, whereas GmMSRB2 and GmMSRB4 were active with the TRX system. Although GmMSRB4 exhibited much lower *k*_cat_ values than GmMSRB2 with either *N*-acetyl-MetO or free MetO, the apparent *K_m_* values for both substrates were the same, and 15- to 20-fold lower than the reported values for *Arabidopsis* MSRB2 [8]. In addition, we observed that the *K*_m_ values of GmMSRB2 and GmMSRB4 for free Met-*R*-O were actually lower than that reported for f*R*MSR from *E. coli* [11]. The catalytic efficiency (*k*_cat_/*K*_m_) of GmMSRB2 was 15- to 150- fold higher than that of any other characterized MSRBs (as reviewed in [41]). In the case of GmMSRB4, the calculated *K*_m_ was low, showing a strong affinity for the substrate, but the low *k*_cat_ led to the question whether or not the yeast TRX regenerated its activity as efficient as it did to GmMSRB2. To clarify this question, we measured the *k_a_* values of yeast TRX2 for GmMSRB2 and GmMSRB4 using *N*-acetyl MetO as substrate. We found the values for GmMSRB2 and GmMSRB4 to be 4.54±0.46 and 2.27±0.51 µM, respectively, suggesting both enzymes can be regenerated by TRX at similar efficiencies.

**Table 2:** Kinetic properties of soybean methionine sulfoxide reductases B.

	*N*-acetyl-MetO			Free *L*-MetO		
	*k_cat_ (s^−1^)*	*K_m_ (µM)*	*k_cat_/K_m_ (M^−1^.s^−1^)*	*k_cat_ (s^−1^)*	*K_m_ (µM)^c^*	*k_cat_/K_m_ (M^−1^.s^−1^)*
GmMSRB1^a^	No activity with either TRX2 or GRX4					
GmMSRB2	2.04±0.06	49±5	42×10^3^	2.86±0.16	2,093±251	1,400
C68S GmMSRB2	No activity					
C121S GmMSRB2	No activity					
GmMSRB4^b^	0.24±0.01	49±6	5×10^3^	0.13±0.01	1,451±239	90
*E. coli* f*R*MSR^d^	–	–	–	6.90±0.40	3,900±400	1,700

Data presented are means ± SE of 3 replicates; a,b; the GmMSRB1 and GmMSRB4 proteins were without the N-terminal signal peptides. c; because the substrate used was a mixture of S- and R- forms, the K_m_ shown is half of the measured values. d; from reference [11].

To confirm the functions of catalytic and resolving Cys, we performed multiple sequence analysis and identified additional conserved Cys at residue 68 (Fig. 1). Site-directed mutagenesis was then performed and two mutants were obtained (C68S GmMSRB2 and C121S GmMSRB2). As shown in Fig. 3, with DTT as the reducing agent, the C68S GmMSRB2 mutant was active towards both protein-based and free MetO with the catalytic parameters similar to those of the wild type enzyme. However, when TRX was used as a reductant, the mutant showed no activity (Table 2), clearly demonstrating a role of Cys68 as a resolving residue. The Cys-to-Ser mutation at residue 121 rendered GmMSRB2 completely inactive toward both substrates using either DTT or TRX as reductants (Fig. 3A,B and Table 2), confirming its role as the catalytic residue.

To test whether the observed activity towards free MetO was taking place in *in vivo*, we performed a complementation assay using an yeast strain whose all three *MSR* genes were knocked out [10]. As shown in Fig. 3C, *GmMSRB2* and *GmMSRB4*, expressed under the control of a strong *GPD* promoter, supported the growth of yeast mutant cells on free MetO as the only source of Met. The level of complementation was similar to that of yeast *fRMSR*, and much stronger than the yeast *MSRB* under the control of the same promoter. It should be noted that, under the control of its own promoter, yeast *MSRB* did not complement at all (Fig. S4). Despite the low calculated *k_cat_* observed with the recombinant GmMSRB4, overexpression of *GmMSRB4* could also provide complementation, suggesting that yeast cells require only a trace amount of free Met to maintain growth. The fact that GmMSRB1 cannot complement the growth of the yeast triple mutant (Fig. 3C) and that yeast TRX2 and GRX4 were unable to regenerate this enzyme *in vitro* (Table 2) implied that GmMSRB1 may require a plant-specific regeneration system.

### Overexpression of GmMSRBs Confers Oxidative Stress Tolerance in Yeast

**Figure 4:**
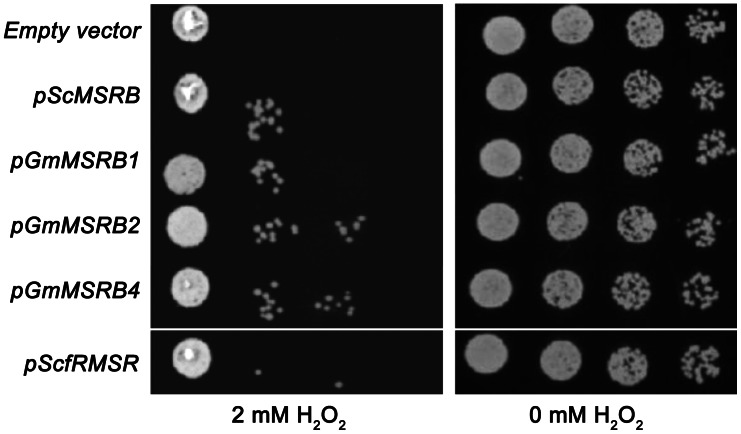
Overexpression of *GmMSRBs* in the *MSR* triple-mutant yeast strain. Yeast cells harboring indicated plasmids (at the OD_600_ of 0.6) were treated with 2 mM H_2_O_2_ for 60 minutes (left), washed to remove excess peroxide and plated. Mock-treated cells were also spotted as a control (right). pGmMSRB1 and pGmMSRB4 did not include sequences encoding signal peptides. Experiment was repeated twice and a typical result is shown.

We previously showed that overexpression of MSRs could protect the yeast strain lacking all three *MSR*s against oxidative stress [10]. Thus, mutant yeast strains overexpressing *GmMSRB*s were tested for their viability in the presence of hydrogen peroxide. As shown in Fig. 4, overexpression of either soybean or yeast *MSRBs* protected cells from H_2_O_2_-induced stress, and the protection was higher in cells overexpressing either *GmMSRB2* or *GmMSRB4*, while overexpressing yeast *fRMSR* alone did not confer significant protection under conditions of our study. Overall, these data suggest that the presence of a GmMSRB possessing a novel activity for free MetO could provide better protection against oxidative stress than MSRBs lacking such activity or f*R*MSR.

Overall, this work reports the discovery of a unique MSRB from soybean that acquired activity for free MetO. This enzyme is as efficient as f*R*MSR both *in vitro* and *in vivo*. We also demonstrate that this enzyme conferred better protection against oxidative stress to yeast cells than either other MSRBs or f*R*MSR. Our work uncovered an unexpected function of MSRBs in plants, which should facilitate research into the roles of MSRs under physiological and pathophysiological conditions as well as potential application in agriculture.

## Supporting Information

Figure S1 **Synteny analysis of soybean GmMSRB genes revealed two segmental duplicated pairs.** Both pairs shared a hug block with 397 anchors. Locus search and image acquisition were done via the web service at http://chibba.agtec.uga.edu/duplication/index/locus.
(PDF)

Figure S2 **Sequence alignments of proteins encoded by different alternate transcripts of GmMSRB2 (upper panel) and GmMSRB4 (lower panel).** Black arrows indicate catalytic Cys, and gray arrows indicate resolving Cys.
(PDF)

Figure S3 **Relative expression of soybean genes encoding MSRB transcripts in various tissues.** Data (normalized reads per million) taken from the cDNA sequencing study by Libault *et al*. (Plant J., 2010, 68∶86–99) [39].
(PDF)

Figure S4 **Complementation assay of the** Δ***3MSR*** **yeast cells transformed with p425-GPD plasmids harboring inidicated yeast** ***MSR*** **s.** The triple mutant was transformed with plasmids and grown on selective media (right panel) or selective media minus *L*-Met and plus *L*-MetO (left panel). In the *pScP::MSRA* and *pScP::MSRB* plasmids, the GPD promoter was replaced with natural yeast promoters for the respective genes.
(PDF)

Table S1 Primers used in this study.
(DOC)
