# Supplementary material for: Diversity of Plant Methionine Sulfoxide Reductases B and Evolution of a Form Specific for Free Methionine Sulfoxide
Source: PLoS One. 2013 Jun 12;8(6):e65637. doi: 10.1371/journal.pone.0065637 (PMC3680461; doi:10.1371/journal.pone.0065637)
Supplement: Figure S1 — Synteny analysis of soybean GmMSRB genes revealed two segmental duplicated pairs. Both pairs shared a hug block with 397 anchors. Locus search and image acquisition were done via the web service at http://chibba.agtec.uga.edu/duplication/index/locus. (PDF) [file pone.0065637.s001.pdf]

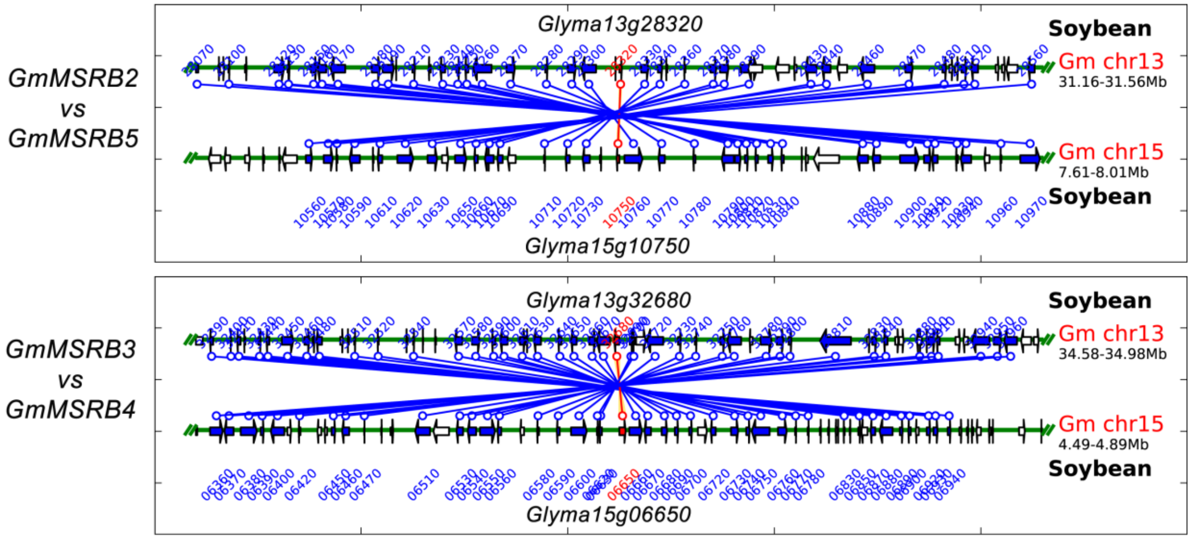

Fig. S1. Synteny analysis of soybean GmMSRB genes revealed two segmental duplicated pairs. Both pairs shared a hug block with 397 anchors. Locus search and image acquisition was done via the web service at http://chibba.agtec.uga.edu/duplication/index/locus.
