# Supplementary material for: Diversity of Plant Methionine Sulfoxide Reductases B and Evolution of a Form Specific for Free Methionine Sulfoxide
Source: PLoS One. 2013 Jun 12;8(6):e65637. doi: 10.1371/journal.pone.0065637 (PMC3680461; doi:10.1371/journal.pone.0065637)
Supplement: Figure S2 — Sequence alignments of proteins encoded by different alternate transcripts of GmMSRB2 (upper panel) and GmMSRB4 (lower panel). Black arrows indicate catalytic Cys, and gray arrows indicate resolving Cys. (PDF) [file pone.0065637.s002.pdf]

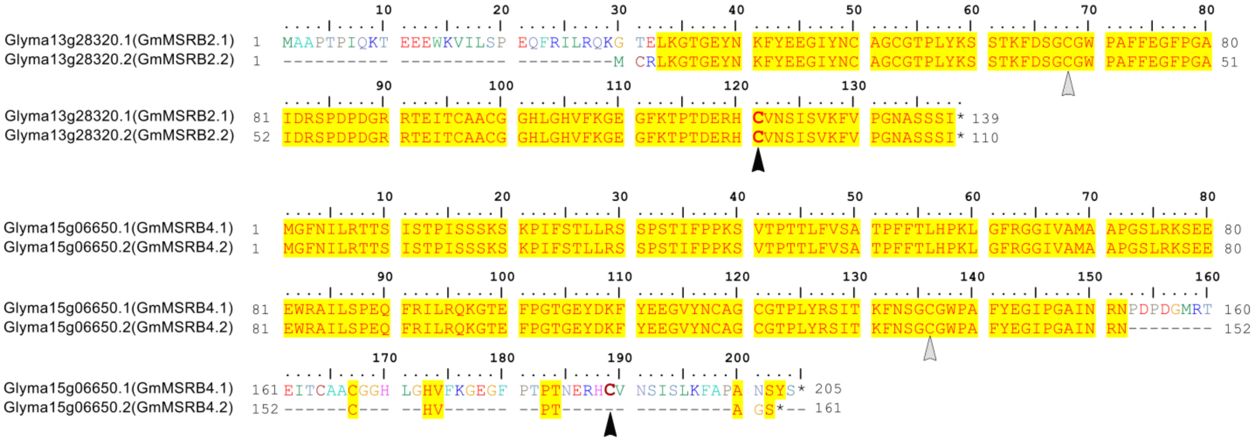

**Fig. S2.** Sequence alignments of proteins encoded by different alternate transcripts of GmMSRB2 (upper panel) and GmMSRB4 (lower panel). Black arrows indicate catalytic Cys, and gray arrows indicate resolving Cys.
