# Supplementary material for: Diversity of Plant Methionine Sulfoxide Reductases B and Evolution of a Form Specific for Free Methionine Sulfoxide
Source: PLoS One. 2013 Jun 12;8(6):e65637. doi: 10.1371/journal.pone.0065637 (PMC3680461; doi:10.1371/journal.pone.0065637)
Supplement: Figure S3 — Relative expression of soybean genes encoding MSRB transcripts in various tissues. Data (normalized reads per million) taken from the cDNA sequencing study by Libault et al. (Plant J., 2010, 68∶86–99) [39]. (PDF) [file pone.0065637.s003.pdf]

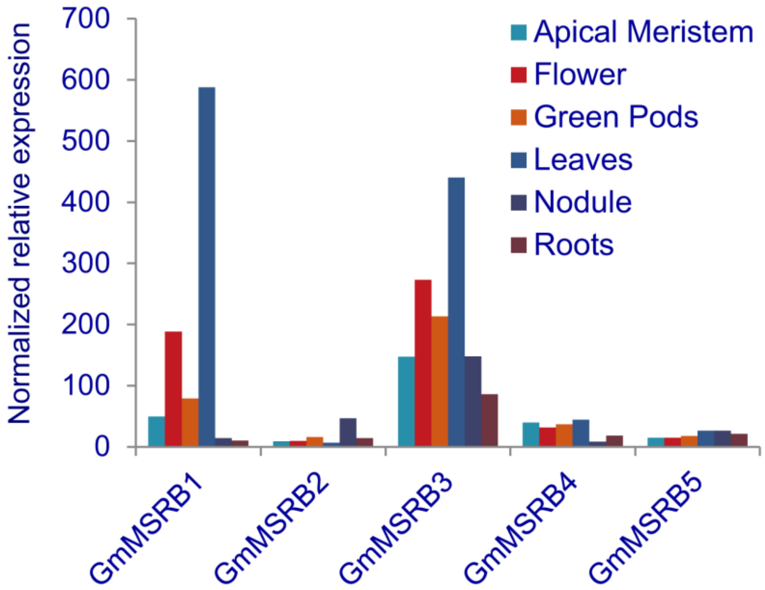

Fig. S3. Relative expression of soybean genes encoding MSRB transcripts in various tissues. Data (normalized reads per million) taken from the cDNA sequencing study by Libault *et al*. (Plant J., 2010, 68:86-99).
