# Supplementary material for: Diversity of Plant Methionine Sulfoxide Reductases B and Evolution of a Form Specific for Free Methionine Sulfoxide
Source: PLoS One. 2013 Jun 12;8(6):e65637. doi: 10.1371/journal.pone.0065637 (PMC3680461; doi:10.1371/journal.pone.0065637)
Supplement: Figure S4 — Complementation assay of the Δ3MSR yeast cells transformed with p425-GPD plasmids harboring inidicated yeast MSR s. The triple mutant was transformed with plasmids and grown on selective media (right panel) or selective media minus L-Met and plus L-MetO (left panel). In the pScP::MSRA and pScP::MSRB plasmids, the GPD promoter was replaced with natural yeast promoters for the respective genes. (PDF) [file pone.0065637.s004.pdf]

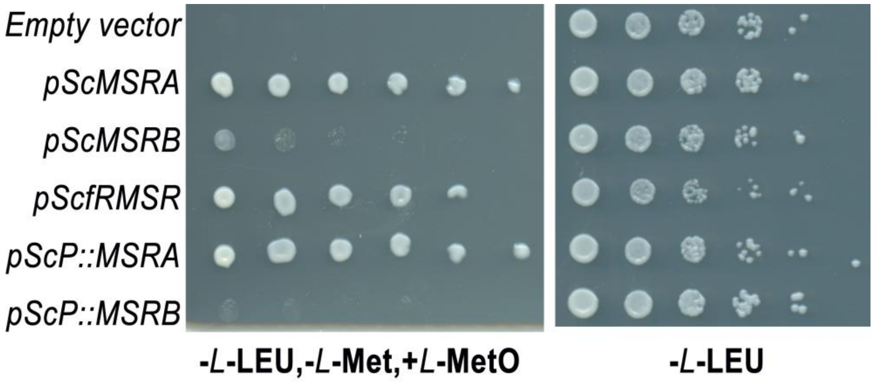

Fig. S4. Complementation assay of the Δ*3MSR* yeast cells transformed with p425-GPD plasmids harboring indicated yeast *MSR*s. The triple mutant was transformed with plasmids and grown on selective media (right panel) or selective media minus *L*-Met and plus *L*-MetO (left panel). In the *pScP::MSRA* and *pScP::MSRB* plasmids, the GPD promoter was replaced with natural yeast promoters for the respective genes.
