# Supplementary material for: Diversity of Plant Methionine Sulfoxide Reductases B and Evolution of a Form Specific for Free Methionine Sulfoxide
Source: PLoS One. 2013 Jun 12;8(6):e65637. doi: 10.1371/journal.pone.0065637 (PMC3680461; doi:10.1371/journal.pone.0065637)
Supplement: Table S1 — Primers used in this study. (DOC) [file pone.0065637.s005.doc]

Table S1. Primers used in this study

| **Attributes** | **Left primers** | **Right primers** | **Vector/Gene** | **Notes** |
| --- | --- | --- | --- | --- |
| GmMSRB1 | CACCATGGCTTCTCAAAGTTTGAGT | CTACTGCTTTGGTTTGAGCTTAAG | pENTR D/TOPO | transfer to pDEST17 |
| GmMSRB2 | CACCATGGCTGCACCAACACCG | TCATATTGAAGAACTAGCATTTCCTG | pENTR D/TOPO | transfer to pDEST17 |
| GmMSRB4 | CACCATGGGCTTCAATATTCTGAGAAC | TTAAGAATAAGAATTGGCTGGCG | pENTR D/TOPO | transfer to pDEST17 |
| GmMSRB1-Nc | AAGGATCCGGAAGCAGGAGCTGATACTATTG | TTCTCGAGCTGCTTTGGTTTGAGCTTAAGG | pET21b | *BamH*I/*Xho*I, truncated at residue S65 |
| GmMSRB4-Nc | AACATATGGCCGCACCTGGCTCTCT | TTCTCGAGAGAATAAGAATTGGCTGGCGC | pET21b | *Nde*I/*Xho*I, truncated at residue A68 |
| GmMSRB2-C68S | CAAGTTTGATTCTGGTTCTGGTTGGCCTGCTTTC | GAAAGCAGGCCAACCAGAACCAGAATCAAACTTG | pDEST17 | Site-directed mutagenesis |
| GmMSRB2-E118A | GACCCAACTGATGCTCGCCATTGTGTCAA | TTGACACAATGGCGAGCATCAGTTGGGTC | pDEST17 | Site-directed mutagenesis |
| GmMSRB2-C121S | CAACTGATGAACGCCATTCTGTCAATAGTATTTCTG | CAGAAATACTATTGACAGAATGGCGTTCATCAGTTG | pDEST17 | Site-directed mutagenesis |
| GmMSRB1 | ACTAGTATGGCTTCTCAAAGTTTGAGT | GTCGACCTACTGCTTTGGTTTGAGCTTAAG | pKS/p425GPD | *Spe*I/*Sal*I |
| GmMSRB2 | ACTAGTATGGCTGCACCAACACCG | GTCGACTCATATTGAAGAACTAGCATTTCCTG | pKS/p425GPD | *Spe*I/*Sal*I |
| GmMSRB4 | ACTAGTATGGGCTTCAATATTCTGAGAAC | GTCGACTTAAGAATAAGAATTGGCTGGCG | pKS/p425GPD | *Spe*I/*Sal*I |
| GmMSRB1-Nc | AAGGATCCATGGCAGGAGCTGATACTATTG | TTCTCGAGCTGCTTTGGTTTGAGCTTAAGG | p425GPD | Yeast, *BamH*I/*Xho*I, truncated at residue S65 |
| GmMSRB4-Nc | AAACTAGTATGGCCGCACCTGGCTCTCT | TTCTCGAGAGAATAAGAATTGGCTGGCGC | p425GPD | Yeast, *Spe*I/*Xho*I, truncated at residue A68 |
| GmMSRB1 | TTCTTCCCACTCCCAGTCAC | TCCCCGGGGTTTTAGTATTC | Glyma08g25610 | qPCR |
| GmMSRB2 | AAGACCCCAACTGATGAACG | TCGATGAAACAGAAAGGCAAG | Glyma13g28320 | qPCR all transcripts |
| GmMSRB3 | AAAGGTGAGGGTGACCAGTG | GGTGGCAGAAACGAAAAGAG | Glyma13g32680 | qPCR |
| GmMSRB4 | ATCCTGATGGGATGAGGACA | CGTCCAAACTGCAGACTCAA | Glyma15g06650 | qPCR all transcripts |
| GmMSRB5 | TTTGTTCCGGGAAGTGCTAC | TGGCCTTGAGACATCAAGAA | Glyma15g10750 | qPCR |
| GmMSRB2.1 | TCCGTCCAAGTTCATTTTCC | CCTCTTCAGTTTTCTGAATCGGT | Glyma13g28320.1 | qPCR |
| GmMSRB2.2 | GAGCATGTGCAGATTGAAGG | CTCAAAGAAAGCAGGCCAAC | Glyma13g28320.2 | qPCR |
| GmMSRB4.1 | CTCTCCCCTGAACAGTTTCG | CCATCAGGATCCGGATTG | Glyma15g06650.1 | qPCR |
| GmMSRB4.2 | CTCTCCCCTGAACAGTTTCG | GGCTGTTGGTACATGACAATTG | Glyma15g06650.2 | qPCR |
| ScTRX2 | AACATATGGTCACTCAATTAAAATCC | AAGGATCCCTATACGTTGGAAGCAATAGC | YGR209C | *Nde*I/*BamH*I |
| ScGRX4 | CCTCCAATACATATGACTGTGGTTGAAATAAAAAGCCAGG | AAAATAAGGCCTCGAGCTGTAGAGCATGTTGGAAATATTCAGG | YER174C | *Nde*I/*Xho*I |
